# *Babesia microti:* an unusual travel-related disease

**DOI:** 10.1186/1471-2334-13-99

**Published:** 2013-02-22

**Authors:** Elodie Poisnel, Mikael Ebbo, Yael Berda-Haddad, Benoit Faucher, Emmanuelle Bernit, Bernard Carcy, Renaud Piarroux, Jean-Robert Harlé, Nicolas Schleinitz

**Affiliations:** 1 Service de médecine interne, AP-HM, Aix-Marseille Université, Marseille cedex 5, 13385, France; 2 Laboratoire d’hématologie, AP-HM, Aix-Marseille Université, Marseille cedex 5, 13385, France; 3 Laboratoire de Parasitologie, AP-HM, Aix-Marseille Université, Marseille cedex 5, 13385, France; 4 Laboratoire de Biologie Cellulaire et Moléculaire Université Montpellier I Faculté de Pharmacie, 15 Avenue Charles Flahault, Montpellier Cedex 5, 134093, France

**Keywords:** Babesiosis, Babesia microti, Hemophagocytosis, Travel-related diseases

## Abstract

**Background:**

Human babesiosis is a rare tick-borne infectious disease. The clinical presentation ranges from an asymptomatic form to a life threatening infection with severe hemolysis. Human babesiosis due to *Babesia microti* is the most common and is endemic in North America.

**Case presentation:**

We report a European patient with severe pancytopenia and reactive hemophagocytosis related to a *Babesia microti* infection. Babesia infection was acquired during a travel in the USA.

**Conclusion:**

Babesiosis should be considered in patients who traveled in endemic areas, especially North America for the most common agent *Babesia microti*.

## Background

Human babesiosis is a tick-borne infectious disease caused by intraerythrocytic protozoan species of the genus *Babesia* transmitted by *Ixodes. Babesia microti* is the most common cause of human babesiosis endemic in USA on the northeastern seabord and the upper midwest. The first confirmed case was a normosplenic individual on Nantucket Island published in 1970 [1]. After additional cases the disease became known as Nantucket fever. The incubation period may last from 1 to 9 weeks and clinical features are similar to those of malaria. The severity is variable depending on the immune status of the host, ranging from an asymptomatic infection to a severe life threatening disease [2]. Severe disease generally occurs in patients over the age of 50 years or with splenectomy, malignancy, HIV, or immunosuppressive medication. *B. microti* infections can also rarely be acquired by transfusion of blood products from asymptomatic donors [3]. In Europe few isolated cases have been reported related to other *Babesia* species: *B. divergens* and *B. venatorum*. Most of European cases are observed in splenectomized patients [2].

## Case report

**Figure 1:**
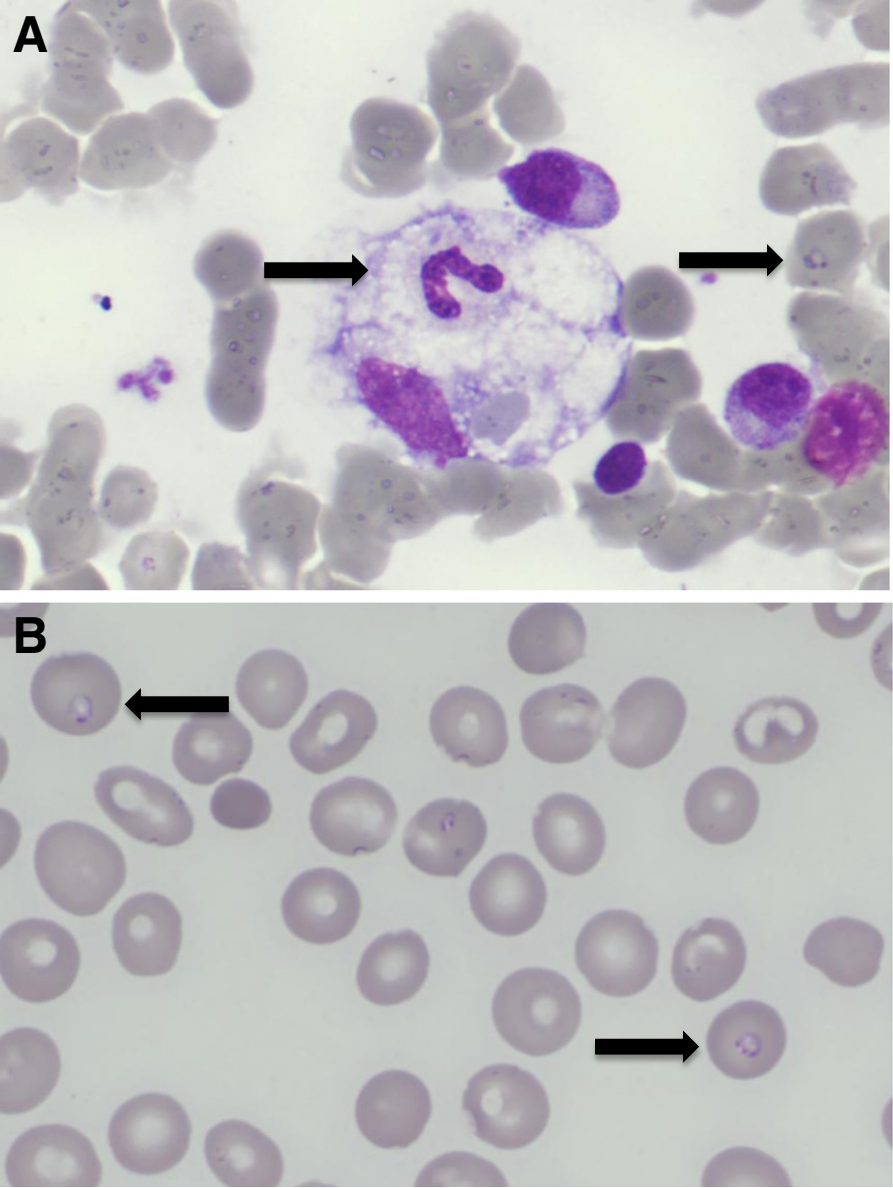
**A; Bone marrow smear stained with Giemsa (magnification x 100).** The picture shows a macrophage with phagocytosis of a neutrophil. On the right side an intra-erythrocytic ring of *Babesia microt*i can be observed in a red blood cell (arrow). **B**; Blood smear stained with Giemsa (magnification x 100) showing intra-erythrocytic forms of *Babesia microti*.

**Figure 2:**
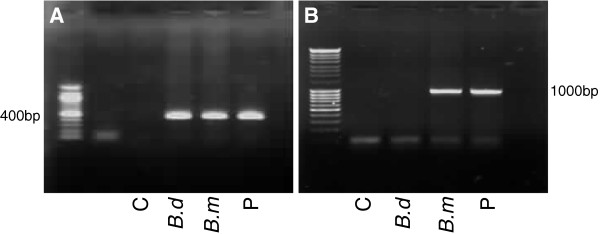
**Polymerase chain reaction results for Babesia microti in patients blood (C=H**_**2**_**0, negative control; B.d= Babesia divergens strain R87, positive control; B.m= Babesia microti strain T, positive control; P=Patient).** PCR was done with specific primers for Babesia [4] in 2**A** and specific amplification of the LDH gene of Babesia microti in 2**B**.

A 82-year-old man living on the French Riviera presented at the emergency department for fever and chills lasting for 5 days. He complained of severe asthenia. He came back two weeks ago from a travel in New York city, USA with a two days stay on the Long Island countryside. He did not note any insect bite during his travel. Jaundice was noticed on examination as well as slightly enlarged spleen and liver. Blood test showed an abnormal blood cell count with neutropenia (0·5 G/L), lymphopenia (0·3 G/L), thrombocytopenia (30×10^9^/L) and anemia (haemoglobin of 91 g/L) with a low reticulocyte count (45 G/L). Blood test also showed increased C reactive protein (154 mg/L), raised ferritin (5953 ng/ml) and liver enzymes were elevated (ALT 56 UI/L (N<40), AST 68 UI/L (N<53), Alkaline phosphatase 213 UI/L (N<129), total bilirubin 48·9 μmol/L). Haptoglobin was undetectable, LDH levels increased 620 UI/L (N<225) and coagulation tests were in normal range. Routine blood cultures were negative. Bone marrow aspiration was performed because of the severe cytopenias associated with a low reticulocyte count and showed typical hemophagocytosis (Figure 1A). Microscopic examination of the blood smear and the bone marrow stained with Giemsa showed intra-eryhtrocytic parasites (Figure 1B). The parasitemia was evaluated about 3% of red blood cells. Rapid testing for Plasmodium falciparum by PCR revealed negative. Intravenous treatment for babesiosis with clindamycin and quinine was started. Patient’s clinical status improved with the resolution of the fever after 3 days of treatment. Blood analysis improved after the 10 days treatment course (haemoglobin 104 g/L, platelets 220×10^9^/L, neutrophils 3 G/L, C reactive protein 16 mg/L). PCR testing on blood for babesia was positive (Figure 2A). *Babesia microti* was confirmed by PCR with specific primers (LDH gene amplification) as shown on Figure 2B. Serology for *Borrelia burgdorferi*, the agent of Lyme disease, was negative.

## Discussion

The clinical symptoms and the biological abnormalities observed during babesiosis are very similar to Plasmodium infection. Thus malaria is often evoked even in travelers who have never visited a malarious area because of the rare “airport malaria” [5]. This case underline that the diagnosis of babesiosis should be considered in patients who traveled in endemic areas, especially North America, for the most common agent *Babesia microti*. *Babesia microti* infection cannot be ruled out in patients with intact spleen in contrary to the European Babesia species (*Babesia divergens* and *Babesia venatorum)*[2]. Moreover in some rare cases spontaneous splenic rupture secondary to *babesia microti* infection have been reported [6]. Babesiosis has been reported to be associated to reactive hemophagocytosis in few cases [7,8] however regenerative hemolytic anemia is the most usual presentation [2]. In the present case the unusual severe neutropenia and non-regenerative anemia led to the bone marrow aspiration analysis. Typical hemophagocytosis was seen however without evidence of phagocytosis of babesia infected red blood cells*.*

Diagnosis of babesia is usually strongly evoked by the blood smear analysis and can be confirmed by specific PCR, allowing to identify *Babesia* species and to exclude plasmodium infection. Despite some similarities with plasmodium, babesiosis does not respond to chloroquine. The use of clindamycin with quinine have been shown to be effective as the combination of atovaquone and azithromycin [2,9]. Because *Borrelia burgdorferi*, the agent of Lyme disease, is transmitted by the same tick and is endemic in North America it should systematically be screened by serology in these patients.

## Conclusion

The diagnosis of babesiosis should be considered in patients who traveled in endemic areas especially North America for the most common agent *Babesia microti*. Blood analyses usually show regenerative hemolytic anemia, thrombocytopenia, and elevated liver enzymes. In case of pancytopenia associated with non-regenerative anemia reactive hemophagocytosis should be evoked. Diagnosis of babesiosis can be made on blood smear analysis but needs usually more specific tests, as PCR, to be confirmed.

## Consent

The patient has given his consent for the publication of this case report.

## Competing interests

The authors declare that they have no competing interests.

## Authors’ contribution

All authors have participated substantially in order to be considered as authors in this case report. All authors read and approved the final manuscript.

## Pre-publication history

The pre-publication history for this paper can be accessed here:

http://www.biomedcentral.com/1471-2334/13/99/prepub
